# Comparison of Pulmonary Functions After Induction of Stress Between Post-COVID and Healthy Adults

**DOI:** 10.7759/cureus.43612

**Published:** 2023-08-16

**Authors:** Syed S Raza, Umema Zafar, Dur E Shehwar, Hunya Amin

**Affiliations:** 1 Department of Physiology, Gajju Khan Medical College, Swabi, PAK; 2 Robert and Suzanne Tomsich Department of Cardiothoracic Surgery, Cleveland Clinic Florida, Peshawar, PAK; 3 Department of Physiology, Khyber Medical University, Peshawar, PAK; 4 Department of Physiology, Rehman Medical College, Peshawar, PAK; 5 Department of Physiology, Khyber Medical College, Peshawar, PAK

**Keywords:** impact of covid-19, 6-minute walk test, pulmonary function tests, stress, covid-19

## Abstract

Since the emergence of COVID in 2019, it has spread worldwide. COVID has affected all the systems of the human body. The present research aimed to assess the effects of COVID-19 on the pulmonary system after stress induction. Healthy and affected individuals between the age of 18 and 40 years were made to perform the 6-minute walk test and their pulmonary functions were compared before and after the stressor. Individuals who were three months post-COVID-19 infection were included as cases. Healthy individuals with no history of COVID were included as controls. The pulmonary functions were performed and noted both at baseline and after the 6-minute walk test. The forced expiratory flow 25 (FEF 25) and peak expiratory flow (PEF) showed statistical significance between both groups (p=0.033 and p=0.007, respectively). FEF 25, 50, and 75, maximum voluntary ventilation (MVV) index, and PEF were positively correlated with all respiratory parameters. Forced expiratory volume % (FEV%) was negatively correlated with vital capacity (VC) and forced vital capacity (FVC). This research helped us establish that the effect on the lungs due to COVID is not due to airway restriction or obstruction but reduced lung volume.

## Introduction

The COVID-19 pandemic has not only hit far but also hard [1]. On 26th February 2020, the first case of COVID-19 in Pakistan was reported in Karachi. Ever since then, Pakistan has encountered four waves of Coronavirus [2]. COVID-19 infection is particularly harmful to the respiratory system in both the short and long term. Far-reaching complications of COVID in patients limit the individual’s performance in particular during stressful situations [3].

The 6-minute walk test (6MWT) is a means of stress induction and is usually carried out to judge the performance of the subjects who have had cardiopulmonary morbidities [4]. It is simple, easy to conduct, better tolerated, and safer than any other exercise tolerance test. 6MWT evaluates the functional capacity of an individual by giving objectivity to the assessment. In doing so, it provides valuable information regarding all the systems during physical activity [5].

Spirometry detects and measures the amount of air a person can take in and out. It can also measure how quickly one can clear the air out of the lungs and also helps in the diagnosis of pulmonary pathologies including asthma and COPD. The British Thoracic Society guidelines, regarding patients with COVID-19 pneumonia, recommended that pulmonary function tests (PFTs) be performed three months after discharge, as even the minor effects of COVID do not diminish before that duration [4,6,7].

The extent to which lung ventilation and oxygenation are affected by COVID depends on the degree of lung damage. The pulmonary infection could completely resolve or result in chronic lung failure and lung fibrosis secondary to fibrogenic molecular pathways [8,9].

Studies lack data on the long-term complications of this medical condition and its effect on pulmonary function [10-13]. PFT is one fine test in this regard that gives objectivity to the degree of COVID infection’s consequences [14].

In the early 70s, it was proposed that the forced expiratory flow (FEF) between 25% and 75% (FEF25-75%) of the forced vital capacity (FVC) be considered a more sensitive tool to test small airway disease. This proposition was based on the evidence analyzed from 53 heavy smokers. It was interesting to note that they did not have asthma and had normal values of FVC, forced expiratory volume in 1 second (FEV1), and FEV1/FVC ratio but an FEF25-75% of less than 80% of the predicted (abnormal) [6].

This research was undertaken to compute the effect of stress (6MWT/exercise) on PFTs in both COVID patients and healthy adults. The baseline pulmonary functions were compared with functions after stress induction. This comparison leads us to determine the effects of COVID on the lungs. Establishing these effects can help us develop precise remedies to counter them. The aim of this research is to assess the effect of the 6MWT on PFTs between healthy and post-COVID young adults of Khyber Pakhtunkhwa.

## Materials and methods

This study was carried out at Khyber Medical College in the Department of Physiology from December 2021 to July 2022 after approval from the Ethical Board of Khyber Medical University (KMU) (protocol code: Dir/KMU-EB/CP/00098, dated 21-12-2021). This is a quasi-experimental trial and consisted of 122 participants. The sample size was determined using G*Power. The participants were enrolled from the main campus of KMU. Flyers were distributed on campus, with contact details of researchers and a preliminary information sheet. Informed written consent was taken from all the people. A detailed proforma was formulated for history taking and to keep a record of participants’ research parameters. One group of 61 were post-COVID cases and the other group of 61 were healthy adults. Subjects aged 18-40 years of both genders were included; those who had COVID previously (three months or more had passed), which was confirmed by PCR test, and who had obvious symptoms that led them to hospitalization were taken as cases. Those who did not have COVID previously or those who had symptomless COVID were taken as healthy adults. Those participants with long-term known complications of COVID, other respiratory diseases such as asthma, COPD, heart disease, uncontrolled hypertension, and physical disability were excluded. Those who were professional athletes and smokers were also excluded. Among the COVID cases, those with respiratory disease, heart disease, hypertension, obesity, and physical disability were excluded. After recruitment, baseline PFTs, oxygen saturation, blood pressure, and heart rate were recorded. The 6MWT is usually performed to judge the performance of the subjects who have had cardiopulmonary morbidities. The subject was asked to walk for 6 minutes (at a pace tolerated by the subject) in a long corridor in laps, and two cones marked the endpoints of the lap. Time was recorded with the help of a standard stopwatch. The subject was seated again after 6 minutes. Oxygen saturation, blood pressure, and heart rate were recorded again on the same subject. Subject PFTs were then measured using a Vitalograph (spirometer) 2 minutes post-stressor. A disposable mouthpiece was given to each subject who was then asked to take a slow breath and blow in the mouthpiece and the subject was asked again to take a fast breath and blow with full pressure in the mouthpiece of Vitalograph (Spiron lab III Vitalograph) for PFT. The variables were fed in SPSS v. 26.0 (IBM Corp, Armonk, NY), and their normality distribution was checked using Shapiro-Wilk tests. The mean and standard deviation were calculated and the t-test was applied for comparison (as all variables were normally distributed). The Pearson correlation was used for finding the correlation level among variables.

## Results

There were 61 subjects each in the cases and healthy adult groups. The mean ± standard deviation (SD) age of healthy adults was 26 ± 5.8 years (females n = 20, 32.8%, and males n = 41, 67.2%). Height had a mean ± SD of 165.6 ± 6.5 cm. Weight had a mean ± SD of 67.3 ± 10.25 kg.

The mean ± SD of age for post-COVID cases was 27.26 ± 6 years (females n = 22, 36.1% and males n = 39, 63.9%). Height had a mean ± SD of 166.27 ± 7.3 cm. Weight had a mean ± SD of 69.18 ± 13 kg. The top three symptoms reported were fever (86.9%), dry cough (73.8%), and body aches (90.2%). Fever, dry cough, and body aches were the most frequent combination of symptoms (57.4%) experienced by the patients. 72.1% consulted a physician for their respiratory illness and 93.4% of the cases got treatment. 

**Figure 1:**
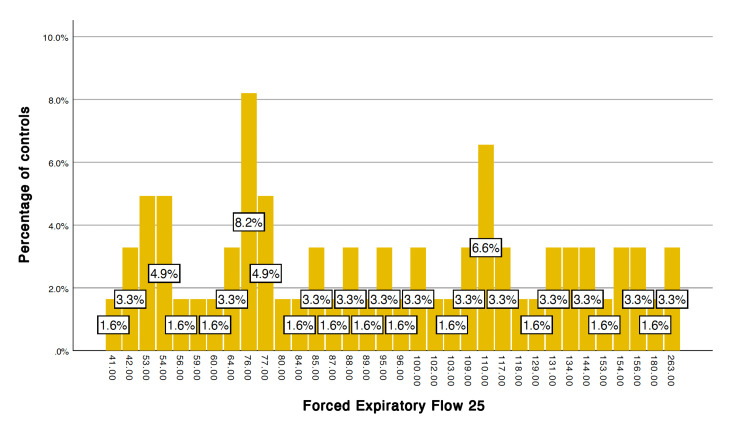
Forced expiratory flow 25 in controls

**Figure 2:**
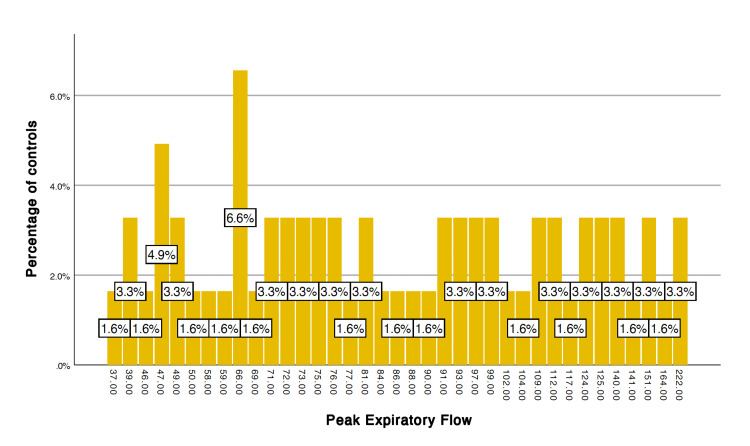
Peak expiratory flow in controls

**Figure 3:**
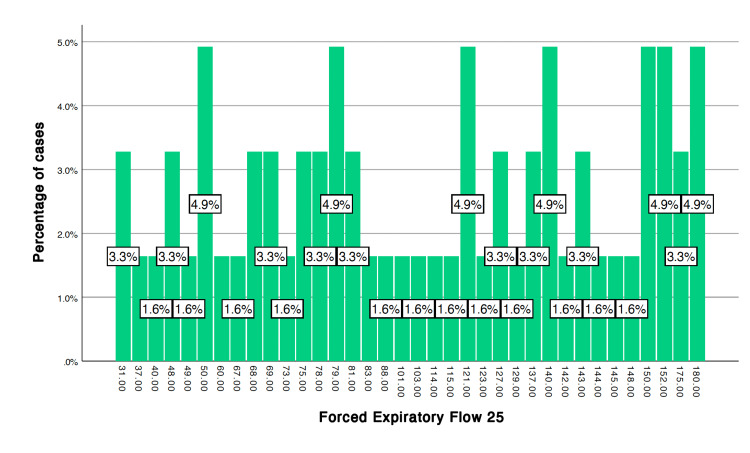
Forced expiratory flow 25 in cases

**Figure 4:**
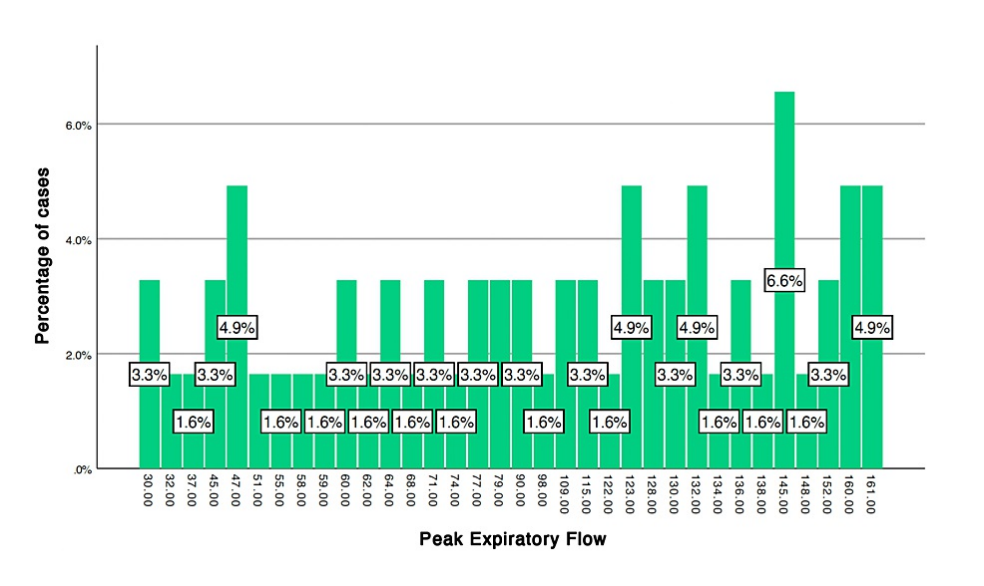
Peak expiratory flow in cases

After applying the unpaired t-test, the following variables showed a significant difference, p < 0.05, between cases and controls after stress induction (6MWT): systolic blood pressure (p = 0.011), heart rate (p = 0.001), FEF 25 (p = 0.033), peak expiratory flow (PEF) (p = 0.007) (Table 1). The frequency distribution of FEF 25 and PEF among the cases and controls is given in Figures 1-4. 

**Table 1:** Post-testing statistics showing statistically significant difference between heart rate, FEF 25, and PEF of cases and healthy adults. The rest of the parameters show no statistically significant difference FEV1, forced expiratory volume in 1 second; FVC, forced vital capacity; FEV%, forced expiratory volume %; FEF, forced expiratory flow; MVV IND, maximum voluntary ventilation index; PEF, peak expiratory flow.

Variables	History of COVID infection	Mean ± Standard deviation	Mean difference	Significance (two-tailed)	95% CI of the mean difference
Oxygen saturation	Cases	98.34 ± 1.33	0.013	0.957	-0.48, 0.51
	Controls	98.32 ± 1.16			
Systolic blood pressure	Cases	121.96 ± 16.23	-8.12	0.011	-14.31, -1.93
	Controls	130.08 ± 14.76			
Diastolic blood pressure	Cases	84.82 ± 15.09	-1.22	0.643	-6.43, 3.99
	Controls	86.04 ± 10.67			
Heart rate	Cases	94.42 ± 15.84	-11.78	0.001	-18.41, -5.16
	Controls	106.20 ± 17.31			
Vital capacity	Cases	103.58 ± 32.94	8.72	0.167	-3.71, 21.16
	Controls	94.85 ± 29.30			
FEV1	Cases	119.74 ± 28.83	4.37	0.446	-6.98, 15.74
	Controls	115.36 ± 28.12			
FVC	Cases	113.02 ± 28.54	1.32	0.831	-10.97, 13.62
	Controls	111.69 ± 32.87			
FEV%	Cases	107.98 ± 6.48	-1.68	0.403	-5.68, 2.30
	Controls	109.67 ± 12.63			
FEF 25	Cases	112.78 ± 40.97	16.33	0.033	1.33, 31.33
	Controls	96.44 ± 33.78			
FEF 50	Cases	110.64 ± 35.72	1.90	0.782	-11.75, 15.56
	Controls	108.73 ± 32.60			
FEF75	Cases	118.38 ± 27.09	-11.51	0.113	-25.8, 2.77
	Controls	129.89 ± 42.91			
MVV IND	Cases	115.29 ± 44.31	8.21	0.338	-8.71, 25.14
	Controls	107.08 ± 40.47			
PEF	Cases	106.98 ± 39.76	19.67	0.007	5.43, 33.92
	Controls	87.30 ± 31.01			

Blood pressure had a positive correlation with heart rate and a negative correlation with PEF and vital capacity. Except for blood pressure (BP), heart rate had no correlation with any other variable. FEF 25, 50, and 75, maximum voluntary ventilation index (MVV IND), and PEF were positively correlated with all respiratory parameters except forced expiratory volume % (FEV%). FEV% was negatively correlated with vital capacity and FVC (Table 2).

**Table 2:** Pearson correlation between variables post-stress show that FEF 25, 50, and 75, MVV IND, and PEF are positively correlated with all respiratory parameters except FEV%. FEV% is negatively correlated with FVC FEV1, forced expiratory volume in 1 second; FVC, forced vital capacity; FEV%, forced expiratory volume %; FEF, forced expiratory flow; MVV IND, maximum voluntary ventilation index; PEF, peak expiratory flow.

Variables	Systolic blood pressure	Diastolic blood pressure	Heart rate	Vital capacity	Post-FEV1	Post-FVC	FEV%	Post-FEF 25	Post-FEF 50	Post-FEF 75	Post-MVV IND	Post-PEF
Oxygen saturation	-0.307^**^	-0.198^*^	-0.056	0.249^**^	0.151	0.047	0.130	0.114	0.116	0.127	0.056	0.080
Systolic blood pressure		0.679^**^	0.191^*^	-0.199^*^	-0.222^*^	-0.081	-0.043	-0.195^*^	-0.155	-0.059	-0.137	-0.187^*^
Diastolic blood pressure			0.243^**^	-0.242^*^	-0.135	-0.130	-0.043	-0.214^*^	-0.193^*^	-0.223^*^	-0.100	-0.213^*^
Heart rate				-0.046	-0.136	-0.053	-0.001	-0.099	-0.019	0.021	-0.081	-0.113
Vital capacity					0.859^**^	0.753^**^	-0.252^**^	0.780^**^	0.652^**^	0.538^**^	0.714^**^	0.794^**^
FEV1						0.897^**^	-0.107	0.764^**^	0.704^**^	0.347^**^	0.844^**^	0.749^**^
FVC							-0.569^**^	0.573^**^	0.514^**^	0.402^**^	0.760^**^	0.588^**^
FEV%								-0.075	0.052	-0.008	-0.142	-0.099
FEF 25									0.838^**^	0.612^**^	0.699^**^	0.971^**^
FEF 50										0.732^**^	0.617^**^	0.778^**^
FEF 75											0.334^**^	0.551^**^
MVV IND												0.710^**^
PEF												
**Correlation is significant at the 0.01 level (two-tailed).												
*Correlation is significant at the 0.05 level (two-tailed).												

The pre-test statistics of those four variables that showed a significant difference (p < 0.05) between post-COVID cases and healthy adults after stress induction were compared (Table 3). It is important to note that heart rate was the only pre-testing variable that showed a significant difference (p < 0.05) between post-COVID cases and healthy adults before stress induction. However, the variables in terms of numerical values showed similar trends for both groups in the pre- as well as post-testing phases (Table 3).

**Table 3:** Pre-testing statistics showing statistically significant difference between the heart rate of cases and controls. The rest of the parameters show no statistically significant difference FEF 25, forced expiratory flow 25; PEF, peak expiratory flow.

Variables	History of COVID Infection	Mean ± standard deviation	Mean difference	Significance (two-tailed)	95% CI of the mean difference
Systolic blood pressure	Cases	119.59 ± 12.11	-4.36	0.057	-8.85, 0.13
	Controls	123.95 ± 12.85			
Heart rate	Cases	84.59 ± 10.99	-4.47	0.037	-8.68, -0.27
	Controls	89.06 ± 12.16			
FEF 25	Cases	106.90 ± 35.86	3.61	0.603	-10.09, 17.31
	Controls	103.29 ± 40.46			
PEF	Cases	102.90 ± 35.58	9.23	0.149	-3.34, 21.80
	Controls	93.67 ± 34.58			

## Discussion

COVID-19 infection is particularly harmful to the respiratory system in both the short and long term. Far-reaching complications of COVID in patients limit the individual’s performance, particularly during stressful situations [3].

Our main findings showed that the following variables showed a significant difference, p < 0.05, between post-COVID cases and healthy adults after stress induction (6MWT): systolic blood pressure (p = 0.011), heart rate (p = 0.001), FEF 25 (p = 0.033), PEF (p = 0.007) (Table 1).

According to Stanojevic, the FEF values have a coefficient of variation (CV) of roughly 20% among adults in comparison to 10% in children. This corresponds to a value of FEF in the range of 60-140% of the predicted. This range/variation/CV broadens as the person ages [15].

**Figure 5:**
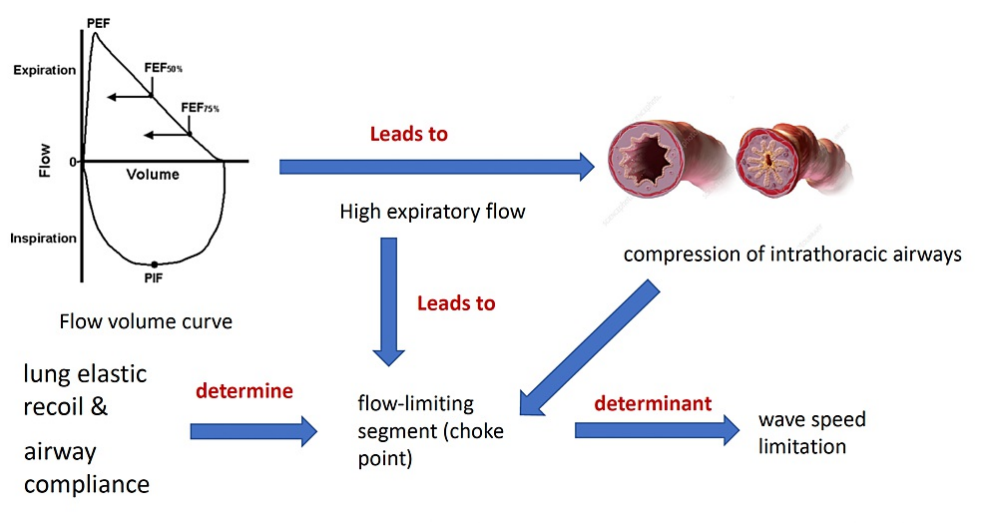
Flow volume curve depicts that high expiratory flow leads to compression of airways which is determined by lung elastic recoil and airway compliance. PEF, peak expiratory flow; FEF, forced expiratory flow.

FEF 25-75% portion of the forced expiratory flow is less effort-dependent than FEV1 and is a measurement of peripheral airway dysfunction. FEF is a more sensitive parameter than FEV1 for evaluating the changes in small airway function in the young population [16,17]. In the course of forced expiration, the raised pleural pressure causes potent intrathoracic airways compression. The site of the flow-limiting segment (choke point) is set on by the interchange between the compliance of the airway and the lung elastic recoil [18-20] (Figure 5).

FEF 25 for cases is 112.78 ± 40.97 while for healthy adults it is 96.44 ± 33.78 with a mean difference of 16.33 (p = 0.033). A recently published study had somewhat similar results showing FEF 25-75 of 85.10 ± 24.05 for cases and 95.35 ± 18.63 for controls. Our study finds out that the PEF for cases is 106.98 ± 39.76 while for healthy adults it is 87.30 ± 31.01 with a mean difference of 19.67, which is in contrast to the study by Salem et al. Their study shows a PEF of 99.00 ± 14.44 for healthy adults and 85.50 ± 23.18 for cases. Another study found a reduction in PEF. Cases had a PEF% range of 86.5-108.5 while controls had a PEF% range of 92-120 [21]. Hence, COVID-19 airway disease has an impact on lung functions, and it causes restrictive lung impairment and alteration of lung function tests [22].

The mechanism causing lung injury/insult by COVID-19 needs further validation and is relatively new to the medical literature. Studies carried out to examine autopsies of COVID-19 patients found that fibrotic changes and microthrombi in the respiratory vasculature were associated with an acute lung insult and diffuse damage to the alveoli [23-25]. 

Another mechanism that could contribute to deteriorating pulmonary function is the fatigue of respiratory muscles. A notable improvement in PFTs was observed in post-COVID patients following six weeks of respiratory rehabilitation. The rehabilitation, however, did not completely recover the patients, suggesting the extent and grit of lung damage [26].

One of the limitations of our study is that diffusing capacity of the lung for carbon monoxide (DLCO) was not tested. A study found that the most common respiratory function derangement was the reduction of DLCO. This was followed by total lung capacity (TLC) and FVC [27,28]. Another limitation was that a follow-up was not performed for the participants.

Furthermore, the included subjects (cases) were mostly infected at the time of the first and second waves of the COVID pandemic. The majority of the patients at that time were either from the older/extreme age group or had comorbidities. In the ongoing fourth wave, this proportion of patients has shifted from older to younger individuals, those who were unvaccinated, and patients with a weak immune response to the vaccine. Therefore, the extent to which the results of this study could be applied to the subjects (cases) of the fourth wave cannot be evaluated. Most of the previous studies evaluating the impact of COVID on PFT have the limitation of the absence of pre-COVID PFT. This limitation was solved by the addition of a healthy adult group to the study design.

Graphical summary

**Figure 6:**
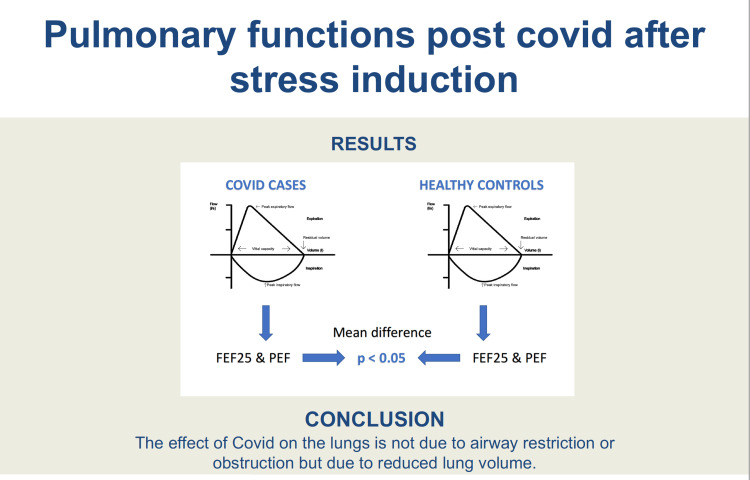
Graphical summary

The graphical summary of the article has been presented in Figure 6.

## Conclusions

Within the limits of this study, it can be concluded that PFTs, particularly FEF 25 and PEF, showed statistical differences between cases and healthy adults (post-stress) and COVID-19 has an effect on these. Reductions in FEF 25 and PEF measurements, in the absence of known airway obstruction (using FEV1/FVC data), result from reduced lung volume and not because of airway disease.
